# A Non-Toxic Concentration of Telomerase Inhibitor BIBR1532 Fails to Reduce *TERT* Expression in a Feeder-Free Induced Pluripotent Stem Cell Model of Human Motor Neurogenesis

**DOI:** 10.3390/ijms22063256

**Published:** 2021-03-23

**Authors:** Virenkumar A. Pandya, Hamish Crerar, Jamie S. Mitchell, Rickie Patani

**Affiliations:** 1 Department of Neuromuscular Diseases, University College London Queen Square Institute of Neurology, Queen Square, London WC1N 3BG, UK; viren.pandya@crick.ac.uk (V.A.P.); hamish.crerar@crick.ac.uk (H.C.); jamie.mitchell.14@ucl.ac.uk (J.S.M.); 2 The Francis Crick Institute, London NW1 1AT, UK

**Keywords:** ageing, amyotrophic lateral sclerosis (ALS), telomerase, TERT, BIBR1532, induced pluripotent stem cells (iPSC), motor neurons (MNs)

## Abstract

Several studies have shown that human induced pluripotent stem cell (iPSC)-derivatives are essentially fetal in terms of their maturational status. Inducing ageing in iPSC-motor neuron (MN) models of amyotrophic lateral sclerosis (ALS) has the potential to capture pathology with higher fidelity and consequently improve translational success. We show here that the telomerase inhibitor BIBR1532, hypothesised to recapitulate the telomere attrition hallmark of ageing in iPSC-MNs, was in fact cytotoxic to feeder-free iPSCs when used at doses previously shown to be effective in iPSCs grown on a layer of mouse embryonic fibroblasts. Toxicity in feeder-free cultures was not rescued by co-treatment with Rho Kinase (ROCK) inhibitor (Y-27632). Moreover, the highest concentration of BIBR1532 compatible with continued iPSC culture proved insufficient to induce detectable telomerase inhibition. Our data suggest that direct toxicity by BIBR1532 is the most likely cause of iPSC death observed, and that culture methods may influence enhanced toxicity. Therefore, recapitulation of ageing hallmarks in iPSC-MNs, which might reveal novel and relevant human disease targets in ALS, is not achievable in feeder-free culture through the use of this small molecule telomerase inhibitor.

## 1. Introduction

An unequivocal consequence of an ageing human population is an increase in age-associated neurodegenerative disease prevalence, including amyotrophic lateral sclerosis (ALS). This is a rapidly progressive degenerative disease of motor neurons (MNs) where patients lose the ability to eat, speak, locomote, and breathe, ultimately causing death within 2–5 years of diagnosis. Normal ageing is the largest risk factor for ALS, yet the interplay between ageing and ALS at molecular [1,2] and cellular levels [3,4,5] remains incompletely resolved in human MNs. With a dearth of disease modifying therapies available, high fidelity modelling is essential to ensure optimal bench-to-bedside translation and therapeutic discovery for ultimate patient benefit.

Patient-specific human induced pluripotent stem cells (iPSCs) can differentiate into progeny from any of the three germ layers via developmentally rationalised directed differentiation paradigms [6], including ectodermally-derived MNs. By maintaining patient disease genetics, the iPSC-MN model can capture disease phenotypes in vitro [7,8,9,10]. During reprogramming, however, iPSCs lose donor markers of ageing, rendering them embryonic and their differentiated progeny of fetal maturational status, regardless of donor age [1,11,12]. It is possible that a failure to integrate ageing into disease models might hamper translational success for neurodegenerative diseases [13].

Various approaches have successfully induced age-related hallmarks in vitro [11,12], including telomere attrition [14]. Telomeres, located at chromosome ends, constitute 6 base pair repeat DNA sequences, which protect against DNA damage. Telomeres shorten with each replicative cycle (and consequently, during normal ageing), with cells ultimately entering a state of cell cycle arrest termed replicative senescence [15]. The enzyme telomerase, absent from most mammalian somatic cells [16] but present in human stem cells, adds telomeric repeat sequences, preventing this shortening [15]. Importantly, telomerase expression is high in iPSCs and consequently they gain telomere lengths comparable with embryonic stem cells [17].

Inhibiting telomerase through the use of the small molecule inhibitor BIBR1532 in human pluripotent stem cells (hPSCs) and their midbrain dopaminergic neuron progeny, revealed age-related phenotypes (dendritic atrophy, DNA damage, mitochondrial perturbation) and Parkinson’s disease-associated changes (loss of tyrosine hydroxylase expression) [14]. We hypothesised that telomerase inhibition via BIBR1532 could also recapitulate age-related hallmarks in iPSC-MNs in feeder-free culture, permitting the interaction between normal ageing and ALS to be modelled with fidelity. We found that the treatment of feeder-free iPSCs with BIBR1532 was cytotoxic across a range of concentrations, which was not rescued by ROCK inhibition (Y-27632). The highest concentration tolerated by iPSCs in culture was insufficient to reduce *TERT* expression throughout differentiation, therefore representing an important limitation in its utility with feeder-free iPSC models.

## 2. Results

### 2.1. A Range of BIBR1532 Concentrations Are Cytotoxic to Feeder-Free iPSCs, and Toxicity Is Not Abrogated by ROCK Inhibition

We sought to establish a telomerase inhibitor treatment paradigm in our highly enriched, comprehensively characterised, and functionally validated iPSC-MN differentiation protocol [7,8,18]. Specifically, cultures were treated for 14 days with BIBR1532 at the pluripotent (iPSC) stage (day −14 to 0) and 18 days during differentiation to MN progenitors (day 0 to 18), after which treatment was removed for terminal differentiation to MNs (day 18 to 25). A negative control arm treated with Dimethyl Sulfoxide (DMSO) was run in parallel (Supplementary Figure S1A). We first investigated whether treatment of feeder-free iPSCs with BIBR1532 at various concentrations was compatible with continued iPSC culture. Previously, 40 µM and 10 µM BIBR1532 had been used to induce telomere attrition in hPSC-derived midbrain dopaminergic neurons [14]. However, treatment of feeder-free iPSCs with 40 µM and 10 µM BIBR1532 was cytotoxic and caused reproducible total cell death when compared to DMSO controls (Supplementary Figures S1B and S2A). Indeed, treatment of iPSCs with various additional BIBR1532 concentrations between 1 µM and 40 µM were similarly cytotoxic to iPSCs (data not shown). However, 40 µM and 10 µM BIBR1532 concentrations were not toxic to human embryonic kidney (HEK293) cells after 48 h, a timepoint by which toxicity was apparent in iPSCs (Supplementary Figure S2A). The small molecule ROCK inhibitor, Y-27632, promotes iPSC survival and growth, possibly via anti-apoptotic and anti-senescent mechanisms [19,20,21]. We hypothesised that co-treatment of iPSCs with 10 µM Y-27632 alongside 10 µM BIBR1532 might alleviate the observed cytotoxicity; however, co-treatment offered no additional protection (Supplementary Figure S1C). The highest concentration of BIBR1532 compatible with continued iPSC culture for the entire 14-day pluripotent stage of BIBR1532 treatment (day −14 to 0) was 0.05 µM (Supplementary Figure S1B).

### 2.2. Treatment of iPSCs with 0.05 µM BIBR1532 Permits Their Directed Differentiation to MNs, but Has No Effect on TERT Expression

**Figure 1:**
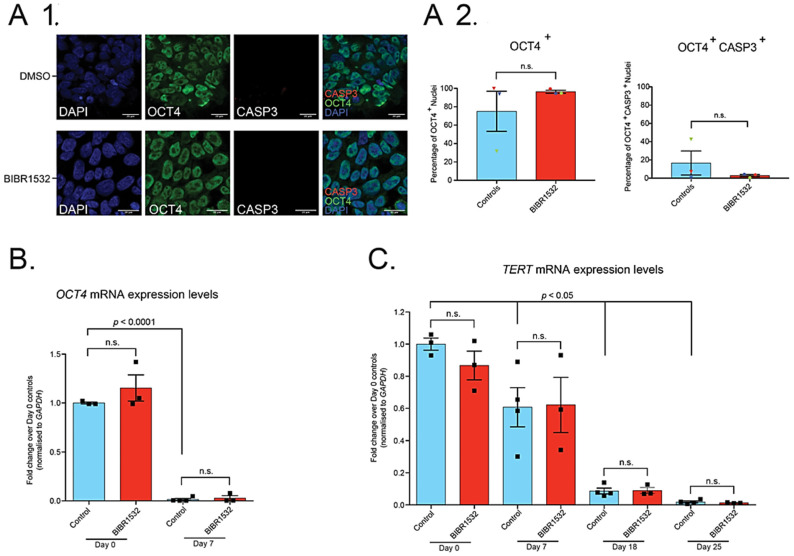
Treatment of iPSCs with 0.05 µM BIBR1532 allows continued culture, maintains pluripotency, and does not induce apoptosis; however, 0.05 µM BIBR1532 has no effect on *TERT* expression throughout iPSC-MN directed differentiation. (**A1**) Qualitative immunocytochemistry for DAPI, OCT4, and activated CASP3 in iPSCs treated with 0.05 µM BIBR1532 or DMSO for 15 days. Scale bars = 20 µm. (**A2**) Quantitative analysis of the percentage of OCT4^+^ nuclei and OCT4^+^CASP3^+^ nuclei in 0.05 µM BIBR1532 treated iPSCs and DMSO controls. Data presented as mean ± S.E.M. Unpaired *t*-test. Three biological repeats (represented by datapoints), five fields per cover slip. (**B**) qRT-PCR analysis of *OCT4* expression at day 0 and day 7 of neural induction, in cells treated with 0.05 µM BIBR1532 or DMSO, normalised over *GAPDH* expression, relative to day 0 control iPSCs. (**C**) qRT-PCR analysis of expression of *TERT* throughout iPSC-MN differentiation in cells treated with 0.05 µM BIBR1532 or DMSO, normalised to *GAPDH* expression, relative to day 0 control iPSCs. (**B**,**C**) Data presented as mean ± S.E.M. One experimental block, three biological replicates. Datapoints represent biological replicates. From day 7 onwards, an additional technical replicate of the Ctrl 1 line was included in controls, represented as an additional datapoint above. Two-way ANOVA, with Tukey’s test for multiple comparisons. n.s. = non-significant, with significance placed at a *p*-value of <0.05. *p*-values displayed graphically refer to the statistical difference between the indicated timepoint and day 0 control samples.

Previous literature had suggested an effect of BIBR1532 treatment on pluripotency [14]. We therefore examined iPSC pluripotency by immunocytochemistry for OCT4, which revealed no difference between 0.05 µM BIBR1532 treated iPSCs and DMSO controls in the percentage of OCT4^+^ nuclei (Figure 1(A1)). There was also no difference between treated iPSCs and controls in the percentage of OCT4^+^ nuclei also positive for the apoptosis marker, activated Caspase 3 (CASP3) (Figure 1(A2)).

Representative phase contrast images of developmental timepoints reveal successful MN differentiation (Supplementary Figure S1D), as expected from our previous studies [7,8,18]. Moreover, qRT-PCR for various developmental markers (*OCT4*, *SOX2*, *NKX6.1*, *TUBB3*) throughout differentiation revealed expected expression changes across time points; however, there was no difference between BIBR1532 treated cells and DMSO controls (Figure 1B, Supplementary Figure S3), suggesting that treatment with 0.05 µM BIBR1532 does not affect iPSC-MN directed differentiation.

Previous literature [22,23,24] has shown that BIBR1532 treatment reduces the expression of *TERT*, a component of the telomerase enzyme complex. We confirmed this in HEK293 cells, which showed a reduction in levels of *TERT* mRNA and protein (Supplementary Figure S2B–D). As previously observed in hPSC-neuronal directed differentiation [14], expression levels of *TERT* mRNA progressively reduced at day 7, 18, and 25 when compared to day 0 iPSCs (Figure 1C). However, qRT-PCR revealed that treatment with 0.05 µM BIBR1532, the highest concentration compatible with continued iPSC culture, had no effect on *TERT* expression across the full range of the developmental time course (Figure 1C).

Taken together, these data demonstrate the highest concentration of BIBR1532 compatible with iPSC survival in feeder-free cultures is not sufficient to affect the telomerase machinery and induce an ageing-like phenotype.

## 3. Discussion

The induction of telomere attrition in vitro via telomerase inhibition has been shown to capture age and Parkinson’s disease-related phenotypes in hPSC-derived midbrain dopaminergic neurons [14]. Telomerase has also been implicated in ALS, with TERC knockout enhancing pathology in ALS mutant mice [25] and TERT expression reduced in post-mortem sporadic ALS patient spinal cords compared to healthy controls [26]. Hence, we hypothesised that recapitulating telomere shortening via telomerase inhibition in iPSCs and their derived MNs might increase the fidelity of modelling MN ageing and ALS.

Using insights from previous literature [14], we established a protocol for telomerase inhibitor (BIBR1532) treatment, involving 14 days at the pluripotent stage and 18 subsequent days during directed differentiation to MNs. However, BIBR1532 treatment led to profound cell death of feeder-free iPSCs at various concentrations between 1 µM and 40 µM after short culture periods. In line with this, BIBR1532 cytotoxicity has been previously reported in various cell types. Indeed, BIBR1532 (25–200 µM) imposed a dose-dependent direct cytotoxic effect and consequent loss of cellular viability in the human glioblastoma cell line, LN18 [22]. Notably, these cells were more sensitive to lower concentrations of BIBR1532 than cells of the immortalised microglial cell line, CHME3, indicating BIBR1532′s toxicity can be cell type-selective [22]. Moreover, BIBR1532 treatment (10–80 µM) displayed dose-dependent toxic effects on proliferation/viability in various blood cancer primary cultures/cell lines, independent of telomerase activity [27]. Viability of non-malignant haematopoietic progenitor cells was unaffected at BIBR1532 concentrations up to 120 µM [27], again indicating differential cell type sensitivity to toxicity. Concentrations above 10 µM BIBR1532 were able to suppress growth and reduce cell viability in a human acute promyelocytic leukaemia (APL) cell line over short culture periods [23]. Given the potential cell type-specific nature of vulnerability to BIBR1532, it might be that iPSCs are also selectively susceptible to toxicity, independent of telomerase inhibition, possibly explaining cell death observed here. However, given previous use of 10 µM and 40 µM BIBR1532 in iPSCs [14], and that lower concentrations than those cited were additionally toxic in this report, this argues for alternative mechanisms of BIBR1532-related cytotoxicity in the present study.

Culture methods might partially explain why iPSCs in this system were more sensitive to low concentrations of BIBR1532. Indeed, whereas iPSCs in our study were feeder-free and cultured in Essential 8 (E8) media, those in the abovementioned study [14] were cultured on mouse embryonic fibroblasts (MEFs) in 20% Knockout Serum Replacement (KSR) media. Direct comparison of media choice (E8 vs. KSR) revealed that hPSCs cultured in E8 possessed nuclear/nucleolar differences, elevated ROS and DNA damage, and higher mitochondrial membrane potential when compared to their KSR-cultured counterparts [28]. Moreover, it is possible that the absence of MEF-secreted supportive factors in feeder-free cultures might render iPSCs more susceptible to toxicity. Future studies might reveal optimised culture conditions to allow feeder-free maintenance of iPSCs treated with previously cited BIBR1532 concentrations. Possible next avenues include supplementing iPSC growth media with MEF-conditioned media, altering media constituents to buffer BIBR1532 toxicity, and developing differentiation paradigms incorporating the benefits of feeder cells transiently.

Given the rapid iPSC death with BIBR1532 treatment we observed here, direct cytotoxicity is a more likely candidate mechanism than telomere attrition-induced senescence. TERT has been shown to possess a number of non-canonical cellular responsibilities, independent of its telomeric functions, including roles in apoptosis inhibition, via antioxidative mechanisms and interaction with reactive oxygen species [29]. In line with this, TERT inhibition via BIBR1532 was shown to increase the percentage of Annexin-V^+^ and Annexin-V^+^/Propidium Iodide^+^ cells, as well as increase Caspase 3 activity in a human pre-B acute lymphoblastic leukaemia cell line [24]. BIBR1532 treatment also induced Caspase 3 activity in a human APL cell line [23]. Given that the ROCK inhibitor, Y-27632, has anti-apoptotic (reduced Caspase 3 activity) [19] and anti-senescent properties [20,21], we hypothesised that it would be a good candidate to abrogate BIBR1532 toxicity in iPSCs. However, co-treatment of iPSCs with 10 µM BIBR1532 and 10 µM Y-27632 failed to rescue iPSCs, suggesting either that toxicity induced by the telomerase inhibitor was so acute that the protective effects of ROCK inhibition were insufficient upon co-treatment or that the mechanism of death was not targeted by ROCK inhibition.

Although there is still controversy, TERT has also been suggested to localise to protect mitochondria and their DNA from oxidative damage in another important non-canonical function [29]. It is thereby additionally possible that TERT inhibition via BIBR1532 might abrogate such protection, leading to toxicity. The combination of culture methods, enhanced susceptibility to apoptosis, and mitochondrial perturbations might together contribute to toxicity seen here, though this is speculative and remains to be experimentally addressed.

Altogether, this report indicates that concentrations between 1–40 µM BIBR1532 are directly cytotoxic to feeder-free iPSCs, and this is not ameliorated by co-treatment with Y-27632. Moreover, the highest concentration of BIBR1532 tolerated (0.05 µM) is insufficient for telomerase inhibition, as evidenced by qRT-PCR analysis of *TERT* expression. To what extent methodological discrepancies between this report and previous literature contribute to the higher cytotoxicity observed here requires further investigation. Nonetheless, by resolving telomere attrition in this model and applying insights from orthogonal methods of inducing in vitro ageing, such as progerin overexpression [12] and transdifferentiation [11], there is potential to enhance the fidelity of human ALS modelling and improve the likelihood of translational success in this devastating disease.

## 4. Materials and Methods

### 4.1. Cell Maintenance and iPSC-MN Directed Differentiation

Cells were housed in humidified incubators (37 °C; 5% CO_2_). HEK293 cells were cultured on Nunc treated plates in DMEM + Glutamax, high glucose medium (Thermo Fisher Scientific 31966021, Waltham, MA, USA) + 10% fetal bovine serum (Sigma F7524) + 1% Penicillin-Streptomycin antibiotics (Thermo Fisher Scientific 15140122). iPSCs and their progeny were cultured on feeder-free Nunc treated plates coated with Geltrex (Thermo Fisher Scientific A1413302). Three biological control lines of iPSCs were used (Ctrl 1–3), detailed elsewhere [7]. iPSCs were cultured in Essential 8 (E8) media (Thermo Fisher Scientific A1517001). Media was changed daily and iPSCs passaged every 2–3 days when 60–90% confluent using 0.5 mM ethylenediaminetetraacetic acid (EDTA) (Life Technologies 15575038, Carlsbad, CA, USA). When required, cells were washed with phosphate-buffered saline (PBS) (Life Technologies 14190144) prior to changing media. ~100% confluency was obtained prior to MN directed differentiation. Neural induction commenced on day 0 in 1:1 N2-B27 (Table S1) + 1 µM Dorsomorphin (Tocris Bioscience 3093, Bristol, UK), 2 µM SB431542 (Tocris 1614), and 3.3 µM CHIR9902 (Miltenyi Biotec 130-104-172, Bergisch Gladbach, Germany). Cells were passaged between day 3–5 using the enzyme dispase at 10 mg/mL (Thermo Fisher Scientific 17105041) and placed in neural induction media + 10 µM ROCK inhibitor (Y-27632; Tocris 129830-38-2) for one day before returning to neural induction media until day 6. From day 7, cells were patterned in 1:1 N2-B27 + 0.5 µM Retinoic acid (Sigma-Aldrich R2625, Saint Louis, MO, USA) + 1 µM Purmorphamine (Merck 540220, Burlington, MA, USA) and passaged between day 11–12 using 10 mg/mL dispase. Cells were in patterning media with 10 µM Y-27632 for one day after passaging, in patterning media until day 13, and in 1:1 N2-B27 + 0.1 µM Purmorphamine until day 18. Cells were optionally expanded in 1:1 N2-B27 prior to terminal differentiation. Cells were passaged using the enzyme accutase (Thermo Fisher Scientific A1110501) and plated on Geltrex + Polyethylenimine (Sigma-Aldrich 408727) in N2-B27 + 10 µM Y-27632, which was replaced one day following passaging by media for terminal differentiation: 1:1 N2-B27 + 0.1 µM Compound E (Enzo Life Sciences ALX-270-415-M001, Farmingdale, NY, USA).

### 4.2. BIBR1532 Experiments

Ctrl 1–3 iPSC lines were passaged using EDTA 15 days prior to neural induction and treated for up to 14 days (survival dependent) at the pluripotent stage with E8 + 0.001-40 µM BIBR1532 (Cambridge Biosciences CAY16608, Cambridge, UK) or DMSO (Sigma-Aldrich D2650) for concentration optimisation. MN differentiation was performed as detailed above, with BIBR1532/DMSO treatment removed after day 18 for terminal differentiation.

### 4.3. Immunocytochemistry

Cells were fixed in 4% paraformaldehyde in PBS (Insight Biotechnology AR1068, Wembley, UK) (room temperature, 12 min), permeabilised, blocked in 5% Bovine Serum Albumin (BSA; Sigma-Aldrich A7030) (room temperature, ≥20 min), and incubated in primary antibody in 5% BSA at room temperature (2 h) or 4 °C (overnight). Following washes in 0.3% PBS-triton (Sigma-Aldrich Triton X-100), cells were incubated for 1 h (room temperature) in secondary antibody (Invitrogen Alexa Fluor, Carlsbad, CA, USA) in 5% BSA. Antibodies and their concentrations are listed in Table S2. Cells were incubated with DAPI (10 min) and then mounted on slides using DAKO mounting media (Agilent Technologies S3023, Santa Clara, CA, USA). Immunocytochemistry was assessed by confocal microscopy, where channels were selected with best signal applied. Laser power and gain were optimised per channel. For qualitative imaging, areas were selected by target staining (averaging 8; bit depth 16). For quantitative analysis, Z stack images were obtained (40× magnification; five fields per cover slip selected by successful DAPI staining; averaging 4; bit depth 8). Channels were split and Z stacks compressed to display maximum intensity immunolabelling (Image J). An automated pipeline was created (Cell Profiler), allowing calculation of the percentage of OCT4^+^ nuclei and percentage of these nuclei that were activated CASP3^+^.

### 4.4. RNA Extraction, cDNA Synthesis, and qPCR

RNA was extracted from snap frozen cell pellets or directly from cells using the Maxwell RSC simplyRNA cells kit (Promega AS1390, Madison, WI, USA) and Maxwell RSC 48 Instrument, as per the manufacturer’s instructions. cDNA synthesis was performed using the RevertAid cDNA synthesis kit (Thermo Fisher Scientific K1621) according to manufacturer instructions and using 5 µM random hexamer primer and 0.3 or 0.65 µg of total RNA in a 20 µL reaction. Negative controls devoid of reverse transcriptase (RT) were run in parallel. qRT-PCR of 10 µL reaction mixtures included 1× PowerUP SYBR Green Master Mix (Applied Biosystems A25778, Waltham, MA, USA), primers at 0.5 µM and 1:20 diluted cDNA. Samples were run in triplicate, alongside RT negative and H_2_O controls, and a melt curve was performed for each reaction. Primer efficiencies were tested using a standard curve of serially diluted cDNA, with an efficiency of ~90–110% accepted. The ddCT method was used to determine relative gene expression levels, which were normalised to *GAPDH* housekeeping gene and in turn to the relevant comparator group (specified in figure legends). Primer sequences are listed in Table S3.

### 4.5. Quantitative Analysis

GraphPad Prism v7.0 was used to perform relevant parametric/non-parametric statistical analyses. Details of statistical testing and replicate numbers are outlined in figure legends.

## Supplementary Materials

The following are available online at https://www.mdpi.com/1422-0067/22/6/3256/s1: Supplementary Figure S1: 10 µM BIBR1532 is cytotoxic to feeder-free iPSCs, not rescued by ROCK inhibition. 0.05 µM BIBR1532 allows continued iPSC culture and is compatible with directed differentiation to MNs, Supplementary Figure S2: 40 µM and 10 µM BIBR1532 is cytotoxic to feeder-free iPSCs, but not HEK293 cells. 40 µM BIBR1532 reduces TERT expression in HEK293 cells, Supplementary Figure S3: 0.05 µM BIBR1532 treatment has no effect on key developmental markers of iPSC-MN directed differentiation, Supplementary Tables: Table S1: N2-B27, Table S2: Antibodies used in this study, Table S3: qRT-PCR primer sequences, Supplementary Materials and methods: Western blotting.
