# Supplementary material for: A Non-Toxic Concentration of Telomerase Inhibitor BIBR1532 Fails to Reduce TERT Expression in a Feeder-Free Induced Pluripotent Stem Cell Model of Human Motor Neurogenesis
Source: Int J Mol Sci. 2021 Mar 23;22(6):3256. doi: 10.3390/ijms22063256 (PMC8005146; doi:10.3390/ijms22063256)
Supplement: Supplementary file 1 [file ijms-22-03256-s001.pdf]

Supplementary Materials

Supplementary Tables

Table S1. N2-B27.

N2		B27	
DMEM-F12 GlutaMAX	Thermo Fisher Scientific 10565018	Neurobasal Media	Thermo Fisher Scientific 12348017
100 μM 2-mercaptethanol	Thermo Fisher Scientific 21985023	B27 supplement	Thermo Fisher Scientific 17504044
100 μM MEM-non essential amino acids	Thermo Fisher Scientific 11140050	2 mM L-glutamine or 1X Glutamax	Thermo Fisher Scientific 25030024<br>Thermo Fisher Scientific 35050061
N2 supplement	Thermo Fisher Scientific 17502048		
5 μg/mL Human Insulin Solution	Sigma-Aldrich I9278		

Penicillin (50 U/mL)/ Streptomycin (50 μg/mL) antibiotics (Thermo Fisher Scientific 15070063)

Table S2. Antibodies used in this study.

Antibody (species)	Reference Dilution	Company	Application
OCT4 (Goat)	1:400	Abcam (Ab27985)	Immunocytochemistry
Cleaved CASP3 (Rabbit)	1:400	Cell Signalling Technology	Immunocytochemistry
Anti-Rabbit IgG, Alexa Fluor 647 (Donkey)	1:5000	Invitrogen (A31573)	Immunocytochemistry
Anti-Goat IgG, Alexa Fluor 488 (Donkey)	1:5000	Invitrogen (A11055)	Immunocytochemistry
Alpha Tubulin (Mouse)	1:1000	Sigma-Aldrich (T5168)	Western blotting
TERT (Rabbit)	1:1000	Abcam (Ab32020)	Western blotting
IRDye® 800CW anti-Mouse IgG (Goat)	1:10000	LI-COR (926-32210)	Western Blotting
IRDye® 680RD anti-Rabbit IgG (Goat)	1:10000	LI-COR (926-68071)	Western Blotting

Table S3. qRT-PCR primer sequences.

Gene	Forward Primer Sequence (5′-3′)	Reverse Primer Sequence (5′-3′)	
GAPDH	ATGACATCAAGAAGGTGGTG	CATACCAGGAAATGAGCTTG	
NKX6.1	GTTTGGCCTATTCGTTGGGGA	GTGCTTCTTCCTCCACTTGGT	
OCT4	H_POU5F1_1	H_POU5F1_1	Sigma
SOX2	H_SOX2_1	H_SOX2_1	Sigma
TERT	TGTGCACCAACATCTACAAG	GCGTTCTTGGCTTTCAGGAT	Vera et al., 2016 [1]
TUBB3	H_TUBB3_1	H_TUBB3_1	Sigma

Supplementary Materials and Methods

Western Blotting

Cell pellets were lysed in RIPA buffer (Thermofisher 89900) + 1× Halt Protease and Phosphatase Inhibitor Cocktail (Thermofisher 78444), sonicated at 4 °C and centrifuged (20,000× *g*; 4 °C; 15 min). The supernatant protein extract was obtained, and concentration determined using the Pierce BCA protein assay kit (Thermofisher 23225) Protein lysates were separated on a NuPAGE 4–12% Bis-Tris protein gel (Thermofisher NP0335BOX) and then transferred onto nitrocellulose membranes. Membranes were blocked at room temperature for 1 hour in 5% milk in PBST. The membrane was incubated in 5% milk in PBST + primary antibody at 4 °C overnight followed by incubation in PBST + 1:10,000 secondary antibody (LICOR IRDye) (room temperature, 1 hr). Membranes were imaged

using the LI-COR Odyssey CLx. Primary antibodies and their concentrations are listed in Table S2.

References for supplementary material

1. Vera, E.; Bosco, N.; Studer, L. Generating Late-Onset Human iPSC-Based Disease Models by Inducing Neuronal Age-Related Phenotypes through Telomerase Manipulation. *Cell Rep.* **2016**, *17*, 1184-1192, doi:10.1016/j.celrep.2016.09.062.
2. Hall, C.E.; Yao, Z.; Choi, M.; Tyzack, G.E.; Serio, A.; Luisier, R.; Harley, J.; Preza, E.; Arber, C.; Crisp, S.J., et al. Progressive Motor Neuron Pathology and the Role of Astrocytes in a Human Stem Cell Model of VCP-Related ALS. *Cell reports* **2017**, *19*, 1739-1749, doi:10.1016/j.celrep.2017.05.024.

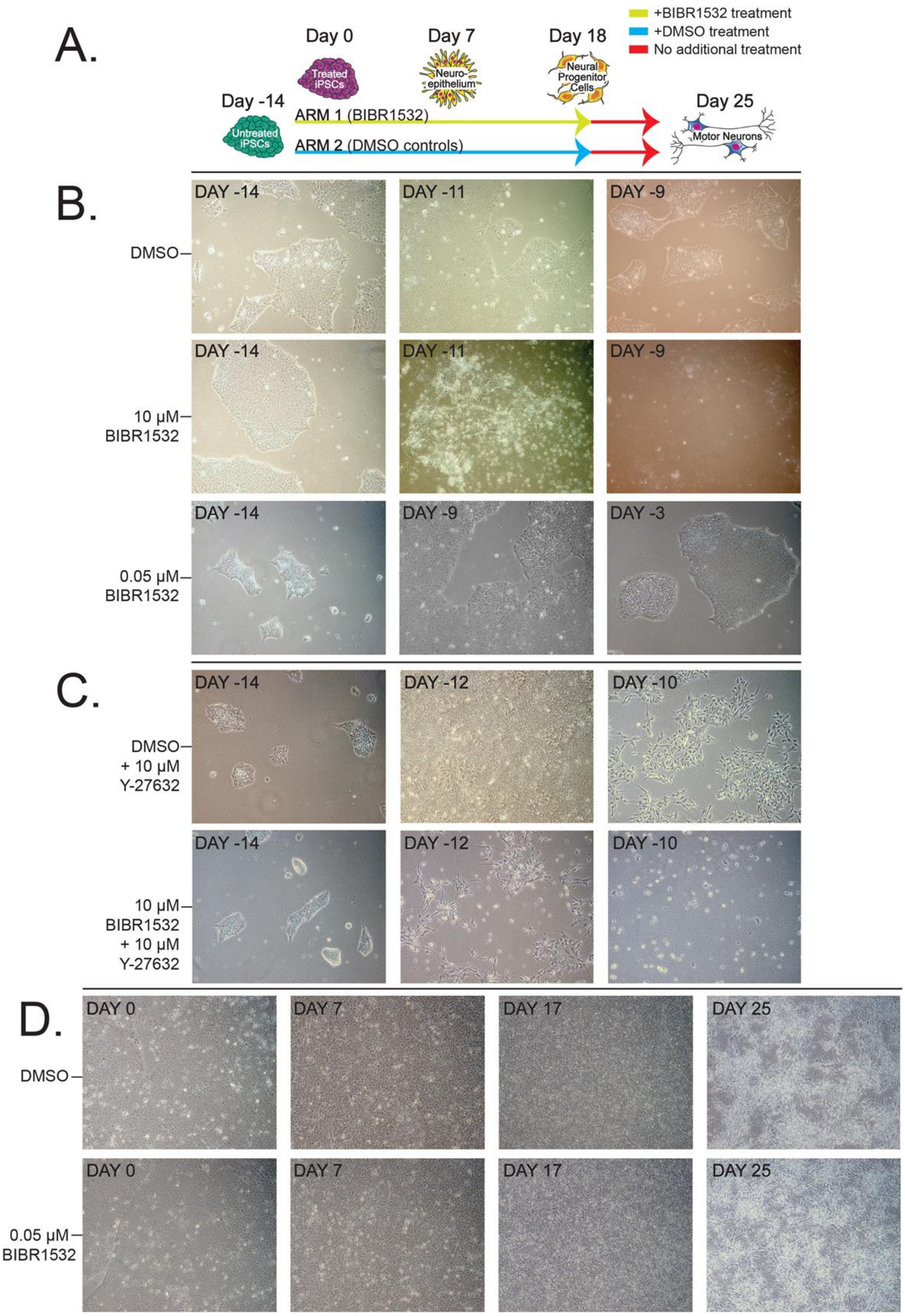

Figure S1. (related to Figure 1): 10 μM BIBR1532 is cytotoxic to feeder-free iPSCs, not rescued by ROCK inhibition. 0.05 μM BIBR1532 allows continued iPSC culture and is compatible with directed differentiation to MNs. (**A**) Schematic depicting experimental paradigm for BIBR1532 treatment and DMSO controls. Figure templates adapted from Hall et al., 2017 [2]. (**B**) Representative phase contrast images of DMSO control iPSCs, and iPSCs treated with 10 μM and 0.05 μM BIBR1532, across time. (**C**) Representative phase contrast images of DMSO control or 10 μM BIBR1532 iPSCs co-treated with 10 μM Y-27632. Note that treatment with Y-27632 changes iPSC morphology. (**D**) Representative phase contrast images of DMSO control and 0.05 μM BIBR1532 treated cells throughout directed differentiation to MNs.

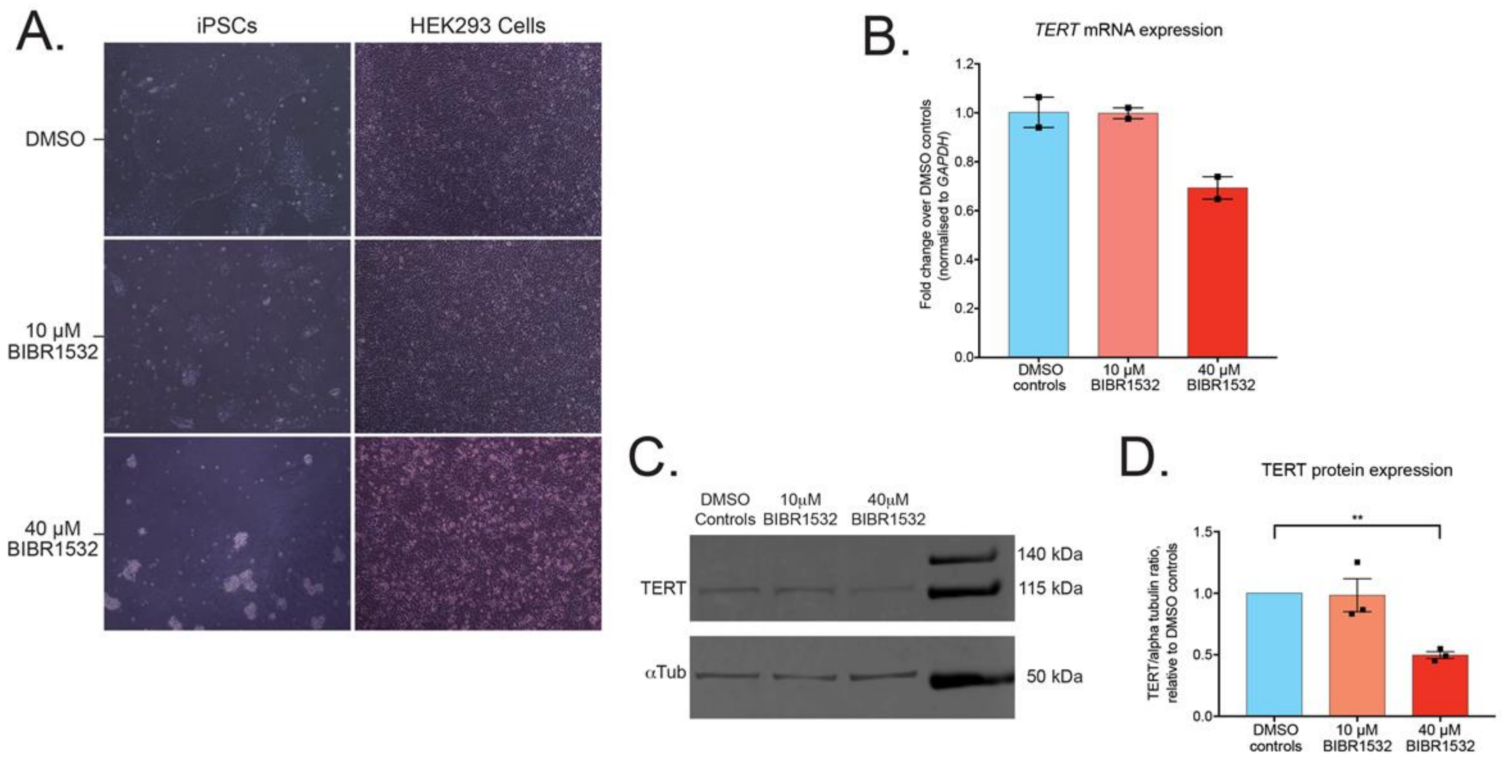

Figure S2. (related to Figure 1): 40 μM and 10 μM BIBR1532 is cytotoxic to feeder-free iPSCs, but not HEK293 cells. 40 μM BIBR1532 reduces TERT expression in HEK293 cells. (**A**) Representative phase contrast images of 40 μM and 10 μM treated iPSCs and HEK293 cells, alongside DMSO controls at 48 hours. (**B**) qRT-PCR analysis of expression of *TERT* in HEK293 cells after 48 hours of 40 μM and 10 μM BIBR1532 treatment, normalised to *GAPDH* expression, relative to DMSO controls. n=2 (represented as datapoints). Data presented as mean ± S.E.M. (**C**) Representative western blot for TERT and alpha tubulin in HEK293 cells after 48 hours of 40 μM and 10 μM BIBR1532 treatment. (**D**) Quantitative densitometry analysis of TERT protein expression, normalised to alpha tubulin protein expression, relative to DMSO controls. n=3 (represented as datapoints). Data presented as mean ± S.E.M. One-way ANOVA, with Tukey's test for multiple comparisons. ** $p < 0.01$.

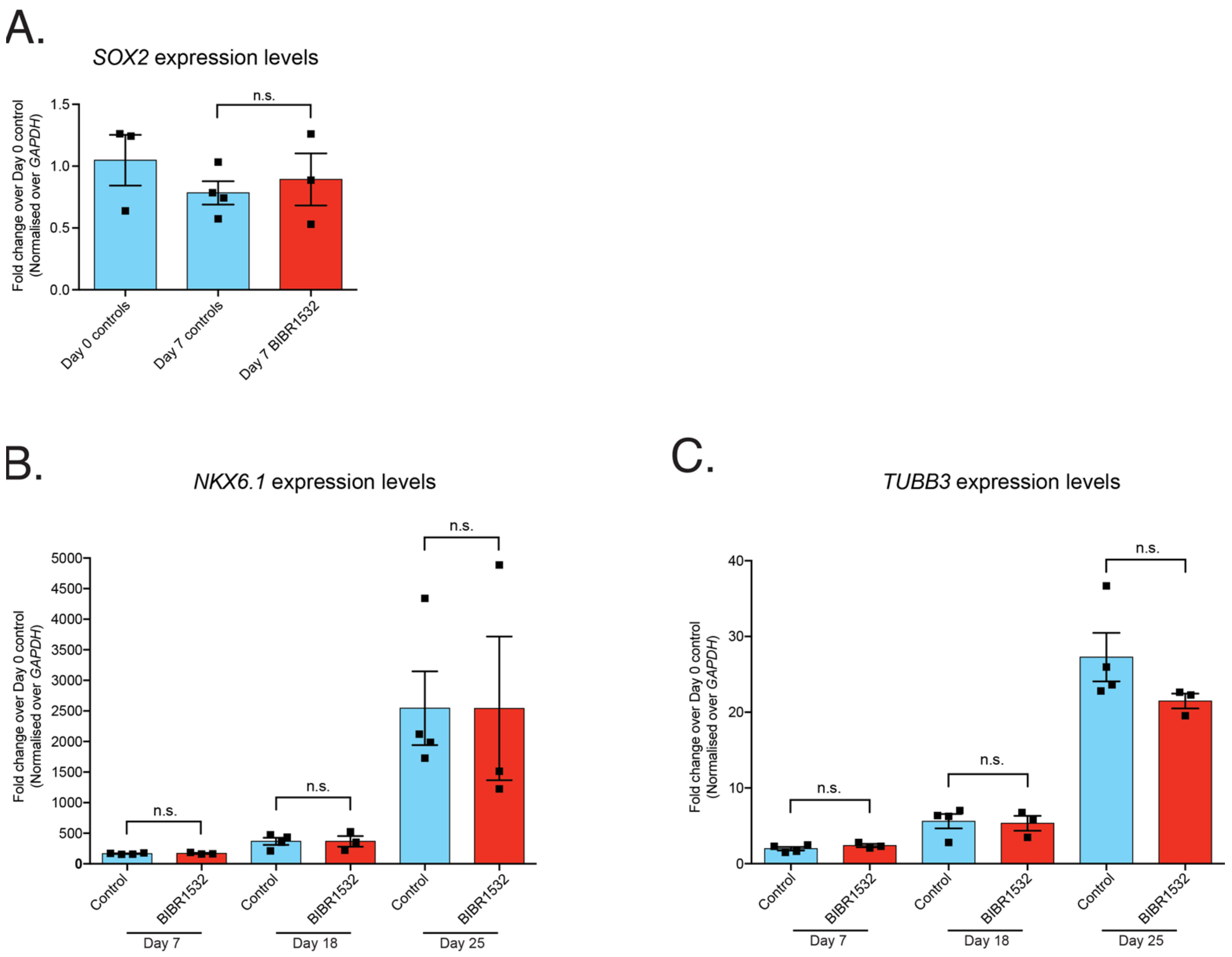

Figure S3. (related to Figure 1): 0.05 µM BIBR1532 treatment has no effect on key developmental markers of iPSC-MN directed differentiation. (**A**) qRT-PCR analysis of expression of *SOX2* in cells treated with 0.05 µM BIBR1532 or DMSO at day 0 and day 7 of iPSC-MN differentiation, normalised to *GAPDH* expression, relative to day 0 control iPSCs. An unpaired t-test of treated/untreated day 7 cells revealed no significant difference in *SOX2* mRNA expression with treatment. (**B**) qRT-PCR analysis of *NKX6.1* expression throughout MN differentiation, in cells treated with 0.05 µM BIBR1532 or DMSO, normalised over *GAPDH* expression, relative to day 0 control iPSCs. Two-way ANOVA, with Tukey's test for multiple comparisons. There was a non-significant two-way interaction between developmental timepoint and BIBR1532 treatment ($p > 0.9999$); there was a statistically significant main effect of timepoint ($p < 0.001$), but not BIBR1532 treatment ($p = 0.9998$). (**C**) qRT-PCR analysis of *TUBB3* expression throughout MN differentiation, in cells treated with 0.05 µM BIBR1532 or DMSO, normalised over *GAPDH* expression, relative to day 0 control iPSCs. Two-way ANOVA, with Tukey's test for multiple comparisons. There was a non-significant two-way interaction between developmental timepoint and BIBR1532 treatment ($p = 0.1667$); there was a statistically significant main effect of timepoint ($p < 0.0001$), but not BIBR1532 treatment ($p = 0.1922$). (**A–C**) Data presented as mean ± S.E.M. 1 experimental block, 3 biological replicates. Datapoints represent biological replicates. From day 7 onwards, an additional technical replicate of the Ctrl 1 line was included in controls, represented as an additional datapoint above. n.s. = non-significant.
